# Southward shift of precipitation extremes over south Asia: Evidences from CORDEX data

**DOI:** 10.1038/s41598-020-63571-x

**Published:** 2020-04-15

**Authors:** Mayank Suman, Rajib Maity

**Affiliations:** 1 School of Water Resources, Indian Institute of Technology Kharagpur, Kharagpur, 721302 India; 2 Department of Civil Engineering, Indian Institute of Technology Kharagpur, Kharagpur, 721302 India

**Keywords:** Climate-change impacts, Hydrology

## Abstract

Analysis of observed Indian Summer Monsoon precipitation reveals more increase in extreme precipitation (in terms of its magnitude) over south India compared to north and central India during 1971–2017 (base period: 1930–1970). In the future, analysis of precipitation from the Coordinated Regional Downscaling Experiment indicates a southward shift of precipitation extremes over South Asia. For instance, the Arabian Sea, south India, Myanmar, Thailand, and Malaysia are expected to have the maximum increase (~18.5 mm/day for RCP8.5 scenario) in mean extreme precipitation (average precipitation for the days with more than 99^th^ percentile of daily precipitation). However, north and central India and Tibetan Plateau show relatively less increase (~2.7 mm/day for RCP8.5 scenario). Analysis of air temperature at 850 mb and precipitable water (RCP4.5 and RCP8.5) indicates an intensification of Indian Ocean Dipole in future, which will enhance the monsoon throughout India. Moisture flux and convergence analysis (at 850 mb) show a future change of the direction of south-west monsoon winds towards the east over the Indian Ocean. These changes will intensify the observed contrast in extreme precipitation between south and north India, and cause more extreme precipitation events in the countries like Myanmar, Thailand, Malaysia, etc.

## Introduction

Changes in the characteristics of extreme precipitation events due to changing climate in the climate “hot-spot” like south Asia^1^ may have far-seeing socio-economical impacts. South Asian region, including the Indian sub-continent, receives most of the precipitation due to monsoon, which is strongly sensitive to climate change^2–6^. The characteristics of Indian Summer Monsoon Rainfall (ISMR) are changing in terms of its trend in extreme precipitation events^7–15^. However, most studies do not agree on the nature of the change. For example, some studies suggest a statistically significant increase in extreme precipitation events post 1980^7–10^ with an increase in the frequency of dry spell and intensity of wet spell^11,12^. However, some studies suggest no significant trend in extreme precipitation across India^13^, and dry spell has a positive trend in most parts of India^14^. Vinnarasi *et al*.^15^ suggested a significant negative trend in wet spell duration and a positive trend in dry spell duration after analyzing large spatial resolution (0.25° latitude × 0.25° longitude) data set. Moreover, the increasing trend in extreme precipitation during the monsoon season is highly localized and vary spatially. The contradicting conclusions about the past state of the Indian monsoon in the literature come from a number of factors that include the choice of data set, study regions, characteristics of extreme considered, and the methodology of the study^15^.

For the future time period, simulations by Coupled Model Intercomparison Project Phase 5 (CMIP5) climate models show an overall wetter monsoon and an increase in extreme precipitation events^16–19^. However, some studies also suggest suppression of Indian monsoon^20^ and a shift in the monsoon onset along with a change in duration^17,20,21^. The CMIP5 simulations reveal that South Asia is expected to receive more, and Central Asia is expected to receive less precipitation than in past^22^. However, the changes in precipitation are not uniform and are governed by localized factors like topography. Most of the above studies used coarse resolution General Circulation Model (GCM) data for analysis, whereas the use of fine-resolution data can help in better understanding the dynamics of monsoon over South Asia^15^.

In this study, spatio-temporal changes in precipitation extremes (if any) for the past and future period are analyzed, and its possible causes are identified for the regions affected by Indian Summer Monsoon (ISM), *i.e*., Indian Ocean, eastern Africa, and south Asia. The study also helps in identifying the most vulnerable regions for extreme precipitation under ISM. First, the observed daily data^23^ over Indian landmass is used to identify the trend, if any, or the change in the spatio-temporal distribution of precipitation extremes between the period 1930–1970 and 1971–2017. These periods are selected based on the climate regime shift in the 1970s. A jump in the mean and 95^th^ percentile of the daily annual precipitation is also noticed over India in the decade of 1970–1980 (please refer to the detailed discussion in the supplementary document). During the study of future precipitation, the areas affected by ISM are included in the study domain. Future precipitation extremes are analyzed using 7 Coordinated Regional Climate Downscaling Experiment (CORDEX) simulations to investigate – whether the behavior of precipitation extremes (their magnitude and spatial pattern) will be similar and, if yes, then what the possible causes are. The details of CORDEX models used in this study are provided in Table S1 (supplementary document). Three characteristics of annual daily precipitation, namely *mean precipitation*, and *threshold (cut-off) and mean precipitation for days with extreme precipitation*, are analyzed for their spatio-temporal changes. Two different thresholds (*i.e*., 95^th^ and 99^th^ percentile; henceforth represented as P95 and P99 respectively) are selected to identify the days with extreme precipitation. The mean of extreme precipitation magnitude equal to or greater than P95 and P99, are denoted by M95 and M99, respectively. Further, the reasons for the changes in the aforementioned precipitation characteristics are investigated by analyzing the air temperature, moisture flux, and moisture convergence from the observed data and future CORDEX simulations.

## Analysis of observed ISMR

The trends in daily precipitation, annual mean daily precipitation, mean daily precipitation during monsoon (June-September) and post-monsoon (October-November), and annual extreme daily precipitation thresholds (P95 and P99) are assessed for statistical significance for two different periods, that are 1930–1970 and 1971–2017 respectively at 5% level of significance. The areas having significant trend are shown in Fig. S2 in the supplementary document. Compared to the period 1930–1970, the characteristics of precipitation have changed in the Indian mainland. For example, the trend of daily precipitation in most parts of the country, including north-east, central and south India, was negative during 1930–1970. However, during 1971–2017, the trend in daily precipitation is found positive in most of the parts in south India. During the period 1971–2017, barring a few cases, a decrease in area under negative trend is observed for annual mean precipitation, P95 and P99. The areas showing increasing trend are less but are concentrated in south India, especially coastal regions.

**Figure 1:**
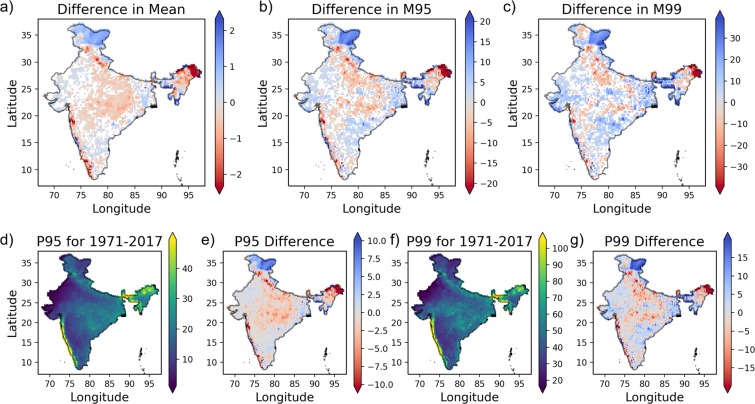
Observed ISMR characteristics. Difference in (**a**) mean daily precipitation, (**b**) M95, and (**c**) M99 (in mm/day) between 1971–2017 and 1930–1970. (**d**) 95^th^ percentile of observed precipitation (in mm/day) during 1971–2017 and (**e**) its difference with respect to 1930–1970. Similar figures (**f,g**) for P99. Colored patches inside Indian landmass in figures (**a–c**) are having statistically significant change.

The difference in precipitation characteristics like mean daily precipitation, and mean precipitation magnitude for days having extreme precipitation (*i.e*., M95 and M99) between 1971–2017 and 1930–1970 are checked for significance using Welch’s $t$-test at 5% level of significance (Fig. 1a–c; results are shown for regions having significant change only). In about 37.3% of the Indian landmass (mostly in the western coast, central and north-eastern part of the country), the mean daily precipitation is found to decrease. However, the mean daily precipitation is found to increase in the eastern coastal region, north-western region, Himalayan region, and some parts of north-eastern states (*i.e*., about 25.2% area).

The P95 and P99 for the period 1971–2017 and its difference when compared to the period 1930–1970 are shown in Fig. 1d–f. The difference in P95 shows a decrease in significant parts of the country (*i.e*., 59.7% area). The P95 has increased in most parts of the eastern coast, Jammu and Kashmir, Ladakh, and some parts of north-east India (*i.e*., 39.9% of the study area). However, the P99 increased in 54.6% area and decreased in 45.2% area of the country. The range of the difference in the case of P99 is higher as compared to P95. Barring few places in central, north and north-eastern India (~28.3% of the study area), the M95 has increased in major parts of the country (~37.3% area; Fig. 1b). Compared to M95, more area (~41%) is showing significant increase in M99 precipitation magnitude (Fig. 1c). About 18.3% area (mostly in north and north-east India) is showing significant decrease in M99 precipitation magnitude. Further, in the case of M95 and M99, the highest observed changes are in the range of ±20 and ±30 mm/day.

Overall, relatively more areas are showing significant changes in M95 and M99 compared to that in the case of mean precipitation. Furthermore, the changes in mean extreme precipitation show different spatial characteristics than the change in mean precipitation. The areas showing increase in the mean extreme precipitation are mostly concentrated in south India (south of 20°N), especially the eastern coast, and some parts of north India (Jammu and Kashmir, Ladakh, Gujarat, and Uttarakhand). On the other hand, the areas showing a decrease in the mean extreme precipitation are concentrated in the central, north and north-eastern India. These contrasting characteristics are more pronounced in the case of mean extreme precipitation compared to mean daily precipitation. These observations suggest that characteristics of ISMR have changed over Indian landmass in 1971–2017 as compared to 1930–1970.

CORDEX model simulations are analyzed for studying the evolution of this contrasting behavior of the extreme precipitation between south and north India in the future. Moreover, It should be noted that the effect of ISMR is not limited to Indian landmass. ISM develops due to the reversal of wind over the equatorial Indian Ocean, and it causes precipitation all over south Asia. Hence, using data from an extended area, including south Asia and the Indian Ocean, may help in getting better insights about the changes in ISM.

## Analysis using CORDEX data for future of precipitation extremes

**Figure 2:**
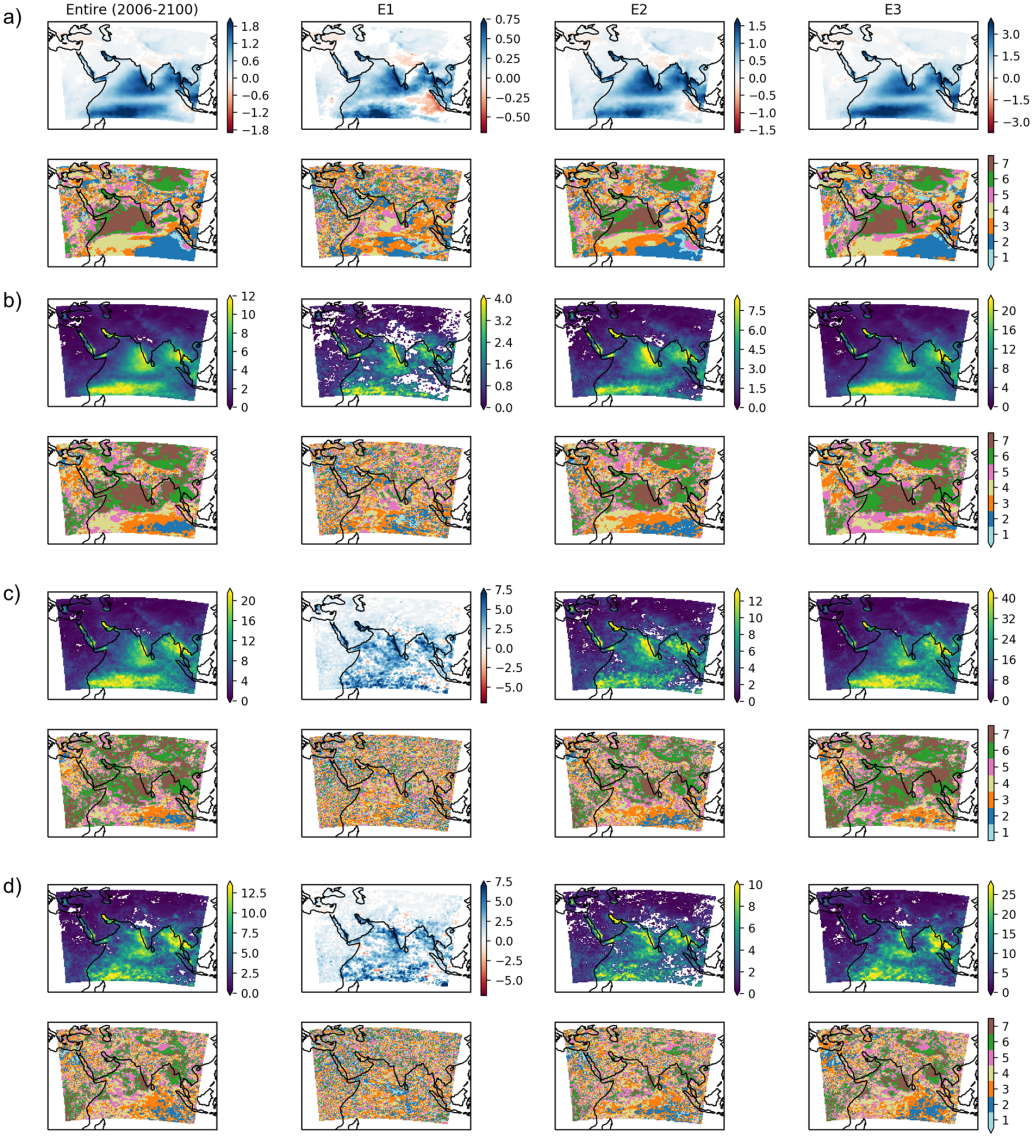
Characteristics of ensemble mean precipitation under RCP8.5 and RCP4.5. Difference between future and historical (1961–2005) precipitation (mm/day) (**a**, top row) in mean, (**b**, top row) in M95, and (**c**, top row) in M99 for RCP8.5. Four columns indicate the entire future period (2006–2100) and three epochs: E1 (2006–2035), E2 (2036–2070), and E3 (2071–2100). (**d**, top row): Same as (**c**, top row) but for RCP4.5. Areas showing no significant change are shown in white patches. Different epochs are studied apart from the entire future period to investigate the temporal variation of change. (**a-d**, bottom row): Spatial distribution of the number of CORDEX models in agreement with the nature of change, i.e., increasing, decreasing, or no significant change.

Mean values from seven CORDEX models (hereinafter ensemble mean; details of CORDEX models are provided in Table S1 in the supplementary document) outputs are analyzed for the change of precipitation over South Asia. The ensemble mean of the CORDEX model outputs is also analyzed for its efficacy in reproducing the past observed precipitation field over the Indian landmass, as discussed in the supplementary document (Fig. S4 in the supplementary document), which is found satisfactory. The CORDEX data may have bias; hence, any statistic for the future simulation (after 2006) is studied with respect to the deviation from the same statistic derived from CORDEX simulated historical values (1961–2005). The data for two scenarios – RCP4.5 and RCP8.5, are available from all selected CORDEX models. These RCPs describe the possible development of greenhouse emission and land-use changes. For instance, the RCP8.5 scenario is the most pessimistic scenario assuming no climate policy, high population growth, and high anthropogenic release of greenhouse gases by 2100. In the case of RCP4.5, the anthropogenic greenhouse gas release is expected to get stabilized by 2100. Hence, the effect of higher anthropogenic activity, specially higher anthropogenic greenhouse gas release, should be evident when comparing changes between RCP8.5 and RCP4.5. However, the uncertainty involved and assumption of individual RCP should be taken into mind^24–27^. Ensemble mean precipitation is analyzed for statistically significant change (if any) in three precipitation characteristics namely mean daily precipitation, M95 and M99 using Welch’s $t$-test at 5% level of significance (Fig. 2 for the ensemble mean of CORDEX model outputs; Fig. S6–S8 for three additional CORDEX models in the supplementary document). Figure 2 also shows the number of CORDEX models in agreement with the corresponding change in precipitation characteristics of ensemble mean CORDEX (same sign of statistical significance). The white patches inside the study area mark the regions having no significant change.

The mean daily precipitation during the entire future period (2006–2100) for RCP8.5 is projected to increase in most parts of south Asia and the Indian Ocean (~84.2% of the study area). However, it is expected to decrease slightly (~0.2 mm/day on an average) in the western Asia, parts of the Indian Ocean near Indonesia, and in the north and central India (~6.5% area of the study area). Most of the models agree with the expected increase in mean daily precipitation over the Indian Ocean for the entire future period. However, when the change in mean daily precipitation is analyzed epoch wise, two important inferences are drawn – (i) the agreement between the models during the first epoch (E1) is not good, which improves in the later epochs, and (ii) the change will intensify in magnitude, and the spatial extent of significant increase in mean daily precipitation will increase in later epochs. For instance, the area under significant increase in the mean daily precipitation is expected to increase from 66.2% of the study area to 83.3% between epoch E1 and E3. Details about percentage area showing the significant change in the different precipitation characteristics along with the magnitude of change are provided in Table S2 in the supplementary document.

**Figure 3:**
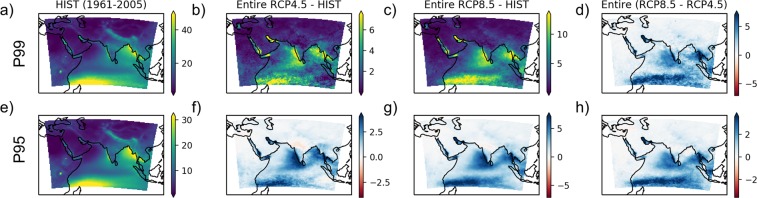
Ensemble mean P99 and P95 (in mm/day) for future (2006–2100) as compared to historical period (1961–2005). (**a**) P99 for historical period. (**b**) Change in P99 in future as compared to historical values for RCP4.5 scenario (**c**) same as (**b**) but for RCP8.5 (**d**) Difference of P99 for RCP8.5 and RCP4.5 scenario (**e–h**) Same as (**a–d**) but for P95.

The P99 and P95 for the historical period and its estimated change in future are shown in Fig. 3a–c, e–g. The figure indicates the increase in P95 and P99 over a vast region of the Arabian Sea, south India, and south-east Asia. Change in extreme precipitation threshold are alarming, for instance, in the case of RCP8.5 at some locations, the P95 and P99 show an increase of more than  $\ge 5$ mm/day and  $\ge 12$ mm/day, respectively.

The M95 and M99 for RCP8.5 scenario and M99 for RCP4.5 scenario are found to increase in most of the study area when the entire future period (2006–2100) is compared with historical period (Fig. 2b–d). For instance, M95 is found to statistically increase over 98.42% area when the entire future period is compared against historical period for RCP8.5 scenario (in the case of epochs, during E1 74.35% is showing significant increase compared to 98.92% in E3). In the case of M99, both the change in precipitation magnitude and area under significant increase is more than M95. When compared epoch wise, the agreement between models is not good for E1; however, it improves for later epochs. In the case of M99, some areas even show decrease (like north and central India, and eastern part of the Indian Ocean near Indonesia) during epoch E1; however, these areas show an increase in M99 by the end of the century. The areas showing decrease in M99, exhibit no significant change in M95 during E1. From Fig. 2c–d, the areas exhibiting the highest increase in mean extreme precipitation include south India, south-east Asian countries like Myanmar, Thailand, Malaysia, etc. and western part of the Indian Ocean in the southern hemisphere. The M99 in these areas shows an increase in the order of  $\ge 40$ mm/day for RCP8.5 and  $\ge 25$ mm/day for RCP4.5 as compared to historical period. These areas are also found to have a higher change in extreme precipitation threshold (P95 and P99) as expected (Fig. 3d–c, f–g). While taking average spatially across all the grid cells in these areas exhibiting statistically significant increase and temporally over the entire future period (2006–2100), the increase in M99 is observed to be 18.5 mm/day for the RCP8.5 scenario.

In the context of south India, one important difference between observed data and CORDEX simulations is noticed. In the case of observed data, the locations on the eastern coast of south India are predominantly showing significant increase (Figs. 1 and S3 in the supplementary document). At some scattered locations on the western coast, a decrease in the mean extreme precipitation was noticed for 1971–2017 (as compared to 1930–1970) in the observed data. However, during the future, more increase in mean extreme precipitation is noticed on the western coast as compared to the eastern coast, as reflected through the ensemble mean CORDEX (Fig. 2). However, the increase in the mean extreme precipitation is statistically significant at both the coasts in ensemble mean CORDEX.

In general, land areas north to 30°N (except some parts of Himalayan and Pamir ranges) are having a comparatively less increase in M99 and M95 (Fig. 2b–d). Most notable among the areas having less increase in mean extreme precipitation is central and north India and Tibetan Highlands. For these regions, the mean increase in M99 is found to be 2.7 mm/day for the entire future period. Hence, in South Asia, extreme precipitation is expected to move southward (as highlighted in Fig. 3 also). For India, the contrast between the extreme precipitation in the northern and southern parts will be more prominent. This observation is also supported by observed data from 1971–2017, as discussed in the last section. However, the distribution of extreme precipitation in south India is different in the case of CORDEX and observed data set.

The projected changes in P99 (Fig. 3d) and P95 (Fig. 3h) are expected to get intensified in the case of RCP8.5 when compared to RCP4.5. Comparison of Fig. 2c,d shows epoch wise intensification of M99 in the case of RCP8.5 as compared to RCP4.5, which is consistent among CORDEX models for areas exhibiting high deviation. The comparative increase in P95 and P99 are high in the areas expected to have higher M95 and M99. These observations suggest the role of anthropogenic activities (comparing RCP8.5 and RCP4.5) in the intensification of extreme precipitation in the future.

## Regional variation of extreme precipitation

**Figure 4:**
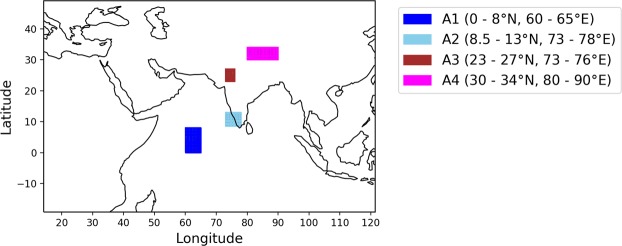
Selected key regions for analyzing the changes in ISMR. The regions are selected based on different characteristics shown by them for change in M99 and M95. For instance, A1 (part of the Arabian Sea) and A2 (part of south India) show a high increase in M95 and M99, and A3 (part of north India) and A4 (Tibetan Plateau) show relatively less increase in M95 and M99.

For analyzing the regional effect of changes in precipitation extremes, four different regions (A1 to A4) are selected (Fig. 4), each having their own distinguishing characteristics. Two regions A1 (part of the Arabian Sea) and A2 (part of south India) are selected due to high increase in M95 and M99. On the other hand, comparatively less increase in M95 and M99 is noticed in the region A3 (part of north India) and A4 (Tibetan Plateau). All the selected regions are associated with ISMR, and, hence, these regions will be helpful in assessing the future changes in ISMR. The regions are studied for different precipitation characteristics like mean daily precipitation, P95, M95, P99, and M99.

The mean precipitation is expected to increase at the locations A1, A2 and A4. While comparing RCP8.5 and historical period, the increase at A1 and A2 ( > 1 mm/day) is comparatively higher as compared to A4 (0.3 mm/day). However, for location A3, the mean daily rainfall is expected to decrease slightly when RCP8.5 is compared to the historical period. It should be noted that the changes are insignificant in many parts of the A3 region in the case of RCP4.5 (Fig. 2a,d). The characteristics for extreme precipitation (*i.e*., P95, P99, M95 and M99) are expected to mostly increase for all locations in the future when compared to their respective historical values (Table 1). However, the higher increase is expected for the locations A1 and A2 as compared to A3 and A4. For instance, in the case of M95 and M99, the expected increase (RCP8.5 vs. HIST) at A2 are found alarming (*i.e*., ~9.7 mm/day for M95 and ~14.5 mm/day for M99) compared to A3 (~0.3 mm/day for M95 and ~1.5 mm/day for M99). In the case of mean precipitation also, the locations A1 and A2 are projected to have a higher increase. Hence, the observations are in agreement with Fig. 2 and highlight the contrasting increase in extreme precipitation between south and north India.

**Table 1:** Different characteristics of precipitation in four key regions.

Characteristics	Period/RCP	Locations*			
		A1	A2	A3	A4
Mean Precipitation (mm/day)	**HIST**	4.19	6.33	1.71	2.38
	**RCP4.5**	4.89	7.49	1.60	2.57
	**RCP8.5**	5.31	8.16	1.65	2.69
P95 (mm/day)	**HIST**	8.29	17.27	6.85	4.54
	**RCP4.5**	9.91	21.59	6.30	5.00
	**RCP8.5**	11.34	24.49	6.65	5.46
M95 (mm/day)	**HIST**	10.01	20.06	9.22	5.33
	**RCP4.5**	12.52	25.59	8.77	5.91
	**RCP8.5**	13.99	29.80	9.52	6.68
P99 (mm/day)	**HIST**	11.04	21.86	10.72	5.86
	**RCP4.5**	13.51	27.71	10.13	6.48
	**RCP8.5**	15.57	32.71	11.02	7.36
M99 (mm/day)	**HIST**	12.78	24.25	13.14	6.51
	**RCP4.5**	17.68	32.24	12.88	7.30
	**RCP8.5**	18.30	38.71	14.66	8.70

HIST represents the period of 1961–2005. RCP4.5 and RCP8.5 represent different scenarios for the period of 2006–2100.

*Refer Fig. 4 for details.

## Possible causes of change in characteristics of precipitation extremes

The increase in precipitation under climate change has been linked to increased water holding capacity of warm air^17,22,28^, changes in moisture flux, and moisture convergence^4,27,29^. ISM is known to be affected by multiple reasons including the difference in air temperature over Tibetan Plateau and Indian Ocean^2,30^, warm moist air over Indian landmass^5,8^ insulated by Himalayas^31^, land-use change^32^, etc. To investigate the possible causes of observed intensification of precipitation extremes, the CORDEX simulations for air temperature at 850 mbar, moisture flux and convergence at 850 mbar, and total precipitable water are analyzed for statistically significant change (if any) between historical and future period. The ensemble means of CORDEX model outputs are used for analyzing the air temperature at 850 mbar, moisture flux, and moisture convergence at 850mbar. However, the output from REMO2009 RCM forced by MPI-ESM GCM is used for the analysis of total precipitable water, as other CORDEX simulations do not provide total precipitable water output. Barring a few regions ($< 0.5 \%$ area), significant increase in temporal mean for all the variables is reported across the study domain when the entire future period (2006–2100) is compared to the historical period (1961–2005) for the RCP8.5 scenario.

**Figure 5:**
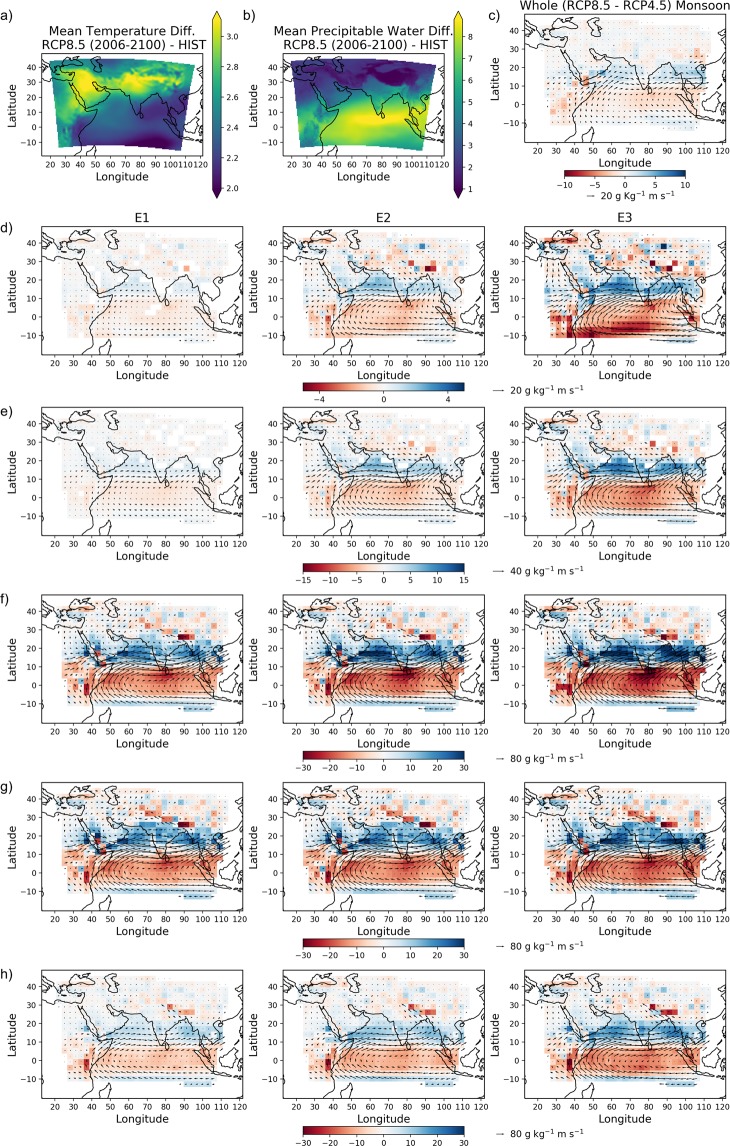
Possible causes of change in precipitation in the future. Difference in (**a**) mean air temperature (in °C) at 850mbar and (**b**) vertically integrated precipitable water (kg/m^2^) between future (2006–2100; RCP8.5) and HIST (1961–2005). (**c**) Difference in mean moisture flux (in g kg^−1^ms^−1^) and moisture convergence (in g/Kg^−1^ms^−1^ per degree) at 850 mbar between RCP8.5 and RCP4.5 scenarios. Difference in mean condition of moisture flux and moisture convergence between different periods in future (RCP8.5) and HIST by considering (**d**) all months (**e**) monsoon months (JJAS). Similarly, difference in mean condition of moisture flux and moisture convergence during days with extreme precipitation ($\ge$ P99) for different epochs and mean condition in HIST at region (**f**) A2, (**g**) A3, and (**h**) A4. For figures **(c–h)** the arrow length and direction show magnitude and direction of moisture flux respectively at 850 mbar. Only the regions with significant changes are shown.

Figure 5a shows the increase in daily mean of air temperature at 850mb over the study domain for the future period. This increase is expected to get higher during epoch E3 (Fig. S9 in the supplementary document). For instance, areas adjoining north India (including the Himalayas and Tibetan Plateau), western Asia show an increase of 5 °C or more for RCP8.5 scenario. The change in mean daily air temperature at 850mb is expected to be more for RCP8.5 as compared to RCP4.5, which indicates the effect of anthropogenic factors, as discussed before. Increased air temperature throughout the study domain will result in an increase in water holding capacity of the atmosphere according to Clausius-Clapeyron relationship^28^, resulting in more extreme precipitation events in general. Being a water tower for the region, air temperature rise in the Himalayan region may possess problem for perennial rivers that are a major source of freshwater in north India. The Arabian Sea region is expected to experience more increase in mean air temperature as compared to sea near Indonesia in the southern hemisphere. These changes are expected to cause intensification of the positive phase of Indian Ocean Dipole^33^.

Similar to air temperature at 850mb, precipitable water content is also found to increase in most parts of the study domain. The comparison between the changes in these climatic variables between RCP4.5 and RCP8.5 scenarios (Fig. S9 and S10 in the supplementary document) shows the effect of anthropogenic factors resulting in their intensification. The increase in precipitable water over the Indian Ocean region is more as compared to the sub-tropics, including Tibetan Plateau. Coupled with the increase in air temperature over Tibetan plateau, it creates favorable conditions for ISM^31,34,35^, which may result in increased extreme precipitation over Indian landmass, as observed in Fig. 2b–d. However, these changes do not provide a plausible explanation for more extreme precipitation in south India compared to its north India (or southward shift of precipitation extreme over South Asia in general).

Analysis of mean annual moisture flux for statistical significant change using Hotelling’s ${t}^{2}$-test at 5% level of significance (Fig. 5d) suggests that the long term changes in moisture flux will result in the intensification of eastward winds over eastern Africa, southern part of the Arabian Sea, south India, and most part of the Bay of Bengal. These changes might be attributed to the intensification of the positive phase of Indian Ocean Dipole^33^. Moisture flux contribution from the Bay of Bengal is projected to increase over south-east Asian countries. Additionally, the moisture flux from the Bay of Bengal over north India is expected to increase as compared to the moisture flux component emanating from the Arabian Sea.

Analysis of future changes in mean moisture flux deviation during monsoon months (June - September; Fig. 5e) suggests an intensification of eastward moisture flux over the Indian Ocean. This intensification carries more moisture-laden wind over south India. Topographical features in the region play a role in the spatially nonuniform distribution of extreme precipitation, as observed in Fig. 2. For instance, the existence of the Western Ghats mountain range causes more precipitation on the windward side (i.e., the western coast regions). This is perhaps the reason for more increase in magnitude of extreme precipitation over the western coast compared to the eastern coast (Fig. 2). The moisture convergence between 10°N and 20°N is found to increase, though not uniformly. These changes in the Arabian Sea will result in higher precipitation over south India; similarly, in the Bay of Bengal, it will result in increased mean daily precipitation over countries like Myanmar, Malaysia, and Thailand. The weaker moisture convergence is noticed over central and north India (in the last epoch – Fig. 5d,e). Additionally, the moisture flux contribution to north India from the Arabian Sea is expected to decrease. The combined effect of these factors might be the reason for either no statistically significant change or small decrease in mean daily precipitation over north and central India (despite the increase in air temperature and precipitable water, and relatively small increase in moisture contribution from the Bay of Bengal in the region) (Fig. 2a). Hence, the moisture flux and convergence deviation during monsoon months are the reason for the changes in mean precipitation over Indian landmass (Fig. 2). From Fig. 5c, more intensified eastward moisture flux, increased moisture convergence between 10°N and 20°N, and weaker moisture convergence at other regions of the Indian Ocean is noticed for the RCP8.5 scenario as compared to RCP4.5 scenario. These observations suggest the role of anthropogenic activities behind the intensification of the eastward shift of moisture flux.

**Figure 6:**
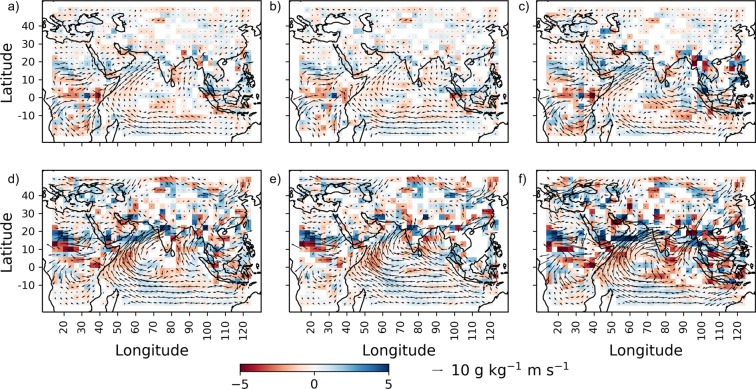
Change in moisture flux and moisture convergence using ERA-Interim data. Difference in mean moisture flux (in g kg^−1^ms^−1^) and moisture convergence (in g/Kg^−1^ms^−1^ per degree) at 850mbar (with respect to 1979 to 2000) for (**a)** 2001 to 2018 (**b)** 2001 to 2010 (**c)** 2011 to 2018 considering data from all months. The figures (**d,e,f)** are similar to (**a,c,d)** but are for monsoon months (JJAS) only. The length and direction of arrows show magnitude and direction of moisture flux, respectively at 850mbar. Only the regions with significant changes are shown.

The future period considered in this study has overlapping duration with data available from ERA-Interim^36^ (2006–2018), hence, similar spatial changes should be noticeable in the ERA-Interim data for this period. The moisture flux and moisture convergence at 850mb obtained from ERA-Interim are analyzed using Hotelling’s ${t}^{2}$-test at 5% level of significance for any significant change between period 2001 to 2018 and 1979 to 2000 (Fig. 6). The increase in eastward moisture flux is noticed in this case too, which intensifies during monsoon months (Fig. 6a,d). Comparing Fig. 6b,c, these changes are strengthening in the near past (2011 to 2018). During monsoon months, the magnitude and eastward shift in moisture flux are found to intensify in the near past (Fig. 6d–f). Furthermore, in the near past, high moisture convergence over most parts of the Arabian Sea is observed during monsoon months (Fig. 6f). Comparing Fig. 6e,f, the moisture contribution to north India from the Arabian Sea is also found to reduce in the near past. The increase in moisture convergence is observed in the northern side of the Deccan Plateau in south India for the same period. With the further intensification of moisture flux over south India, it is expected that more regions with high moisture convergence might be observed in the future, as indicated in Fig. 5e.

Additionally, the analysis of Dipole Mode Index (DMI) for Indian Ocean Dipole calculated from 3-month moving averaging of sea surface temperature does not show any trend. However, its mean and variance are exhibiting a statistically significant increase in the period 1971–2017 when compared to 1930–1970. A two-fold increase in positive IOD post-1970 is also observed when compared to pre-1970 (Section A1.3 and Fig. S11 in the supplementary document). As stated earlier, these changes strengthen monsoon over India.

The difference in mean moisture convergence and the difference in mean moisture flux between the days having extreme precipitation greater than or equal to P99 for RCP8.5 and historical period over region A2, A3, and A4 are shown in Fig. 5f–h. The precipitation extremes over the region A2 mostly occur during monsoon months (June-September). Figure 5f shows that extreme events in this area are caused by increased moisture flux over the western coast of India from the Arabian Sea. Due to the exceptionally high positive moisture convergence deviation over the Indian Ocean region between 10°N and 20°N, the extreme precipitation may become more frequent in the region. Most of the extreme events at the location A3 are in the month of July-August. As observed earlier also, during extreme events at A3 (Fig. 5g), the moisture flux contribution from the Bay of Bengal is higher over north India as compared to historical mean. The extreme precipitation events at A4 are found to occur mostly in the later monsoon period, *i.e*., August and September. Most of the moisture for extreme precipitation at A4 comes from the Bay of Bengal (Fig. 5h). The extreme precipitation events in south-east Asian countries like Myanmar, Thailand, etc. are expected to intensify due to the increase in moisture flux contribution from the Bay of Bengal due east. The magnitude of change in moisture flux on an average is less for extreme precipitation events at A4 as compared to A2 and A3.

In summary, the increased air temperature over Tibetan Plateau and the Himalayas, intensification of IOD, and increased precipitable water over Indian landmass will help in intensification of ISM over the entire Indian landmass. Extreme precipitation is expected to increase all over the study domain by the end of century. For north India, the moisture flux from the Bay of Bengal is expected to increase in future, whereas that from the Arabian Sea is expected to decrease. Additionally, there will be a weakening moisture convergence in north and central India. On the other hand, the moisture flux from the Arabian Sea is expected to increase over south India along with higher moisture convergence in future. Analysis over a larger geographical extent reveals that the precipitation extremes will shift southward over south Asia due to the intense eastward shift of moisture flux between 10°N and 20°N in the Indian Ocean region. The areas expected to have the highest increase in extreme precipitation are the Arabian Sea, south India, and south-east Asian countries like Myanmar, Thailand, and Malaysia. As a consequence, increase of extreme precipitation magnitude in south and north India will exhibit a contrasting feature – more in south Indian region and comparatively less in north and central India.

As a future scope of this study, it may be mentioned that the CORDEX simulations are mostly forced by Green House Gas (GHG) emissions whereas historical changes are governed by different forcings, such as anthropogenic GHG increase, aerosol increase, land-use change, solar insolation changes, natural variability, and others. Thus, a sensitivity analysis of different forcings and moisture budget analysis can be taken up to have more insights into the observed changes in precipitation.

## Methods

For analyzing the trend in different characteristics of observed ISMR (daily precipitation, annual mean of daily precipitation, mean of daily precipitation during monsoon (June-September) and post-monsoon (October-November) and extreme precipitation thresholds (95^th^ percentile - P95 and 99^th^ percentile - P99)), two-sided Mann-Kendall test is used with 5% level of significance. The statistical significance of change in mean daily precipitation, M95 (mean daily precipitation for days having precipitation equal to or above P95), and M99 (mean precipitation for days having precipitation equal to or more than P99) is assessed using two-sided Welch’s $t$-test at 5% level of significance.

Seven CORDEX models utilized in the study have different spatial coverage and resolution (Table S1 in the supplementary document). Hence, to maintain coherency, the outputs of all CORDEX models are regridded to the spatial resolution of 0.5° latitude × 0.5° longitude with the spatial extent of 45.75°N, 19.25°E to 15.75°S, 116.25°E using inverse square distance weighting before any analysis. The ensemble mean precipitation of the seven CORDEX models is calculated. Different characteristics of CORDEX ensemble mean precipitation (mean daily precipitation, P95, P99, M95, and M99) are assessed for significant change using Welch’s $t$-test at 5% level of significance. Additionally, to analyze the regional changes, analysis of future precipitation is carried out at four regions selected on the basis of different characteristics of either mean or extreme precipitation.

Vertically integrated precipitable water from REMO2009 RCM, and air temperature, specific humidity, wind speed in zonal and meridional direction at 850mbar from ensemble mean of CORDEX data are analyzed to find the possible causes of observed changes in future precipitation. Moisture flux at 850 bar vector field (both zonal and meridional components) is obtained as the product of specific humidity and wind speed. The change in air temperature at 850 mb and vertically integrated precipitable water are checked for statistical significance using two-sided Welch’s $t$-test at 5% level of significance. Hotelling’s ${t}^{2}$-test at 5% level of significance is used to assess the statistical significance of change in the moisture flux field. The moisture flux and moisture convergence (convergence of moisture flux field) are regridded to 3° latitude by 3° longitude grid for visualization and analysis of results.

## Supplementary information

Supplementary Information.

## Data Availability

Observed daily ISMR for the period 1930–2017 are obtained from India Meteorological Department (IMD)^23^ (http://www.imdpune.gov.in/Clim_Pred_LRF_New/Grided_Data_Download.html). The data set has a spatial resolution of 0.25° latitude × 0.25° longitude. For the future period analysis, the daily data for precipitation, wind speed in meridional and zonal direction, specific humidity, air temperature and total precipitable water at daily scale are obtained from Coordinated Regional Downscaling Experiment (CORDEX) simulations through the Earth System Grid Federation (https://esgf-node.llnl.gov/projects/esgf-llnl/). Seven different CORDEX model outputs are used in this study. The details of these CORDEX models (like driving GCM, spatial resolution, geographic extent, etc.) are tabulated in Table S1 in the supplementary document. The historical period is assumed to be 1961–2005 (designated as HIST in the main manuscript), and the future period is assumed to be 2006–2100. The future period is further divided into three epochs: • Epoch 1 (2006–2035) also designated as E1 • Epoch 2 (2036–2070) also designated as E2 • Epoch 3 (2071–2100) also designated as E3 Zonal and meridional wind speed at 850mbar, and specific humidity during 1979–2018 is obtained from ERA-Interim project^36^. Observed SST and IOD analysis for duration 1930–2017 is done using NOAA Extended Reconstructed Sea Surface Temperatures version 5 (ERSSTv5) data set^37^. These datasets are used to validate the results obtained from CORDEX data.

## Code availability

The python codes used for post-processing and analysis of IMD, CORDEX, ERSSTv5, and ERA-Interim data are archived, and these are available for research/academic purposes on request from the corresponding author. Additional python libraries are needed to run the code (proper requirements.txt file provided in code).
