# Supplementary material for: Southward shift of precipitation extremes over south Asia: Evidences from CORDEX data
Source: Sci Rep. 2020 Apr 15;10:6452. doi: 10.1038/s41598-020-63571-x (PMC7160141; doi:10.1038/s41598-020-63571-x)
Supplement: Supplementary file 1 — Supplementary Information. [file 41598_2020_63571_MOESM1_ESM.docx]

**Supplementary Information**

Southward shift of precipitation extremes over south Asia: Evidences from CORDEX data

Mayank Suman^1^ and Rajib Maity^2^

^1^School of Water Resources, Indian Institute of Technology Kharagpur, Kharagpur-721302, India

^2^Department of Civil Engineering, Indian Institute of Technology Kharagpur, Kharagpur-721302, India

# A 1.1 Preliminary Analysis

For the study of observed Indian Summer Monsoon Rainfall (ISMR), the study region includes region associated with Indian Summer Monsoon winds with a focus over Indian landmass. To assess the changes in observed Indian Summer Monsoon Rainfall, the study period is divided into two time periods based on reported climate regime shift during 1970s ^1-3^. Additionally, a jump in the mean and extreme annual precipitation over India is also found during 1970s (figure S1). Both mean daily precipitation and 95^th^ percentile annual precipitation (P95) series over India show different trend for pre- and post-1970. In the case of mean daily precipitation, the trends in both periods are significant with 5% significance in Mann-Kendall trend analysis. The mean daily precipitation for India is found to decrease during 1971-2017. However, in the case of P95, the trend was significantly negative before 1970, but after 1970 there is no statistically significant trend in the series. The observed trend for pre- and post-1970 periods are not uniform across the states, as shown by figure S1 c–e.


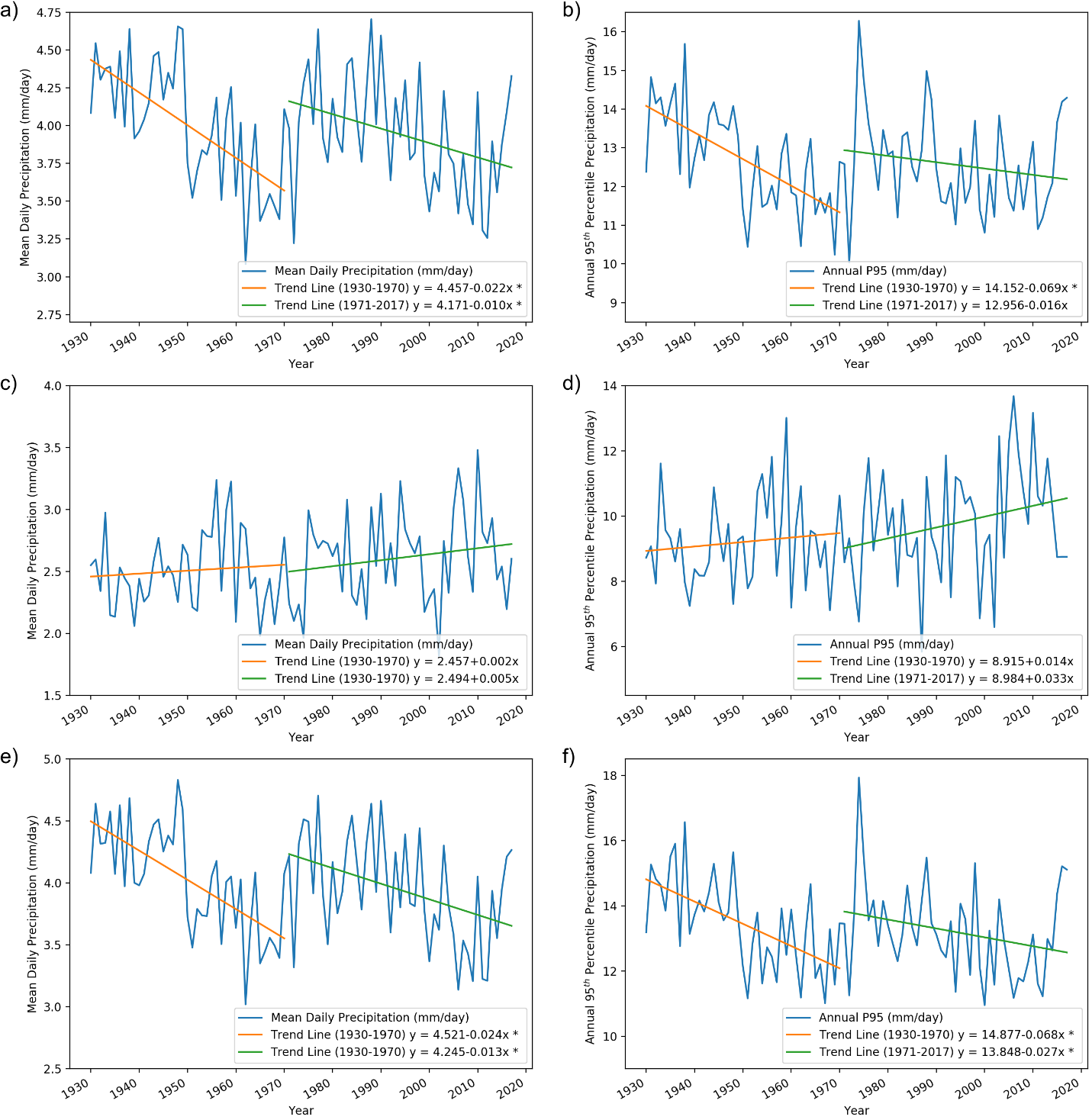


Figure S1: Trend in **a)** mean daily precipitation and **b)** 95^th^ percentile annual precipitation (P95) for India. **c)** and **d)** are similar to **a)** and **b)** but for states having major parts with statistically significant increase in mean precipitation (Figure 1 of the main document; South Indian states, Jammu & Kashmir, Ladakh, Gujarat, and Uttarakhand). **e)** and **f)** are similar to **a)** and **b)** but for states showing mostly decreasing trend in mean precipitation (North India except Jammu & Kashmir, Ladakh and Uttarakhand, and North-East India). The trend equations marked with * show the trend line is statistically significant at 5% significance level using Mann-Kendall trend analysis.

The study of the trend of different characteristics of ISMR (figure S2) and statistical significance analysis for difference in mean and other characteristics of precipitation (figure S3; detail discussion provided in the main manuscript) indicate the spatially varying changes in ISMR in the period 1971-2017 when compared to 1930-1970.

For the better understanding of the changes in ISMR, an extended area associated with ISMR is considered. Hence, eastern part of Africa, entire south Asia, including south-east Asian countries, and the Indian Ocean are included in the study domain to identify the change and its possible cause. The dataset for precipitation, total precipitable water, air temperature, specific humidity, zonal and meridional wind speed at 850mb are obtained from seven CORDEX model simulations for the period 1961-2100. Out of this period, the period of 1961-2005 is considered to be historical simulation, and future estimates under different RCPs are available for 2006-2100. The details of these seven CORDEX simulations are provided in table S1. The Indian Institute of Tropical Meteorology perform six CORDEX model simulations and Climate Service Center, Max Planck Institute for Meteorology performed one CORDEX simulation. As CORDEX simulation may have bias, so all the analysis for future data is done for the deviation of variables from historical means (obtained from historical CORDEX data from period 1961-2005).


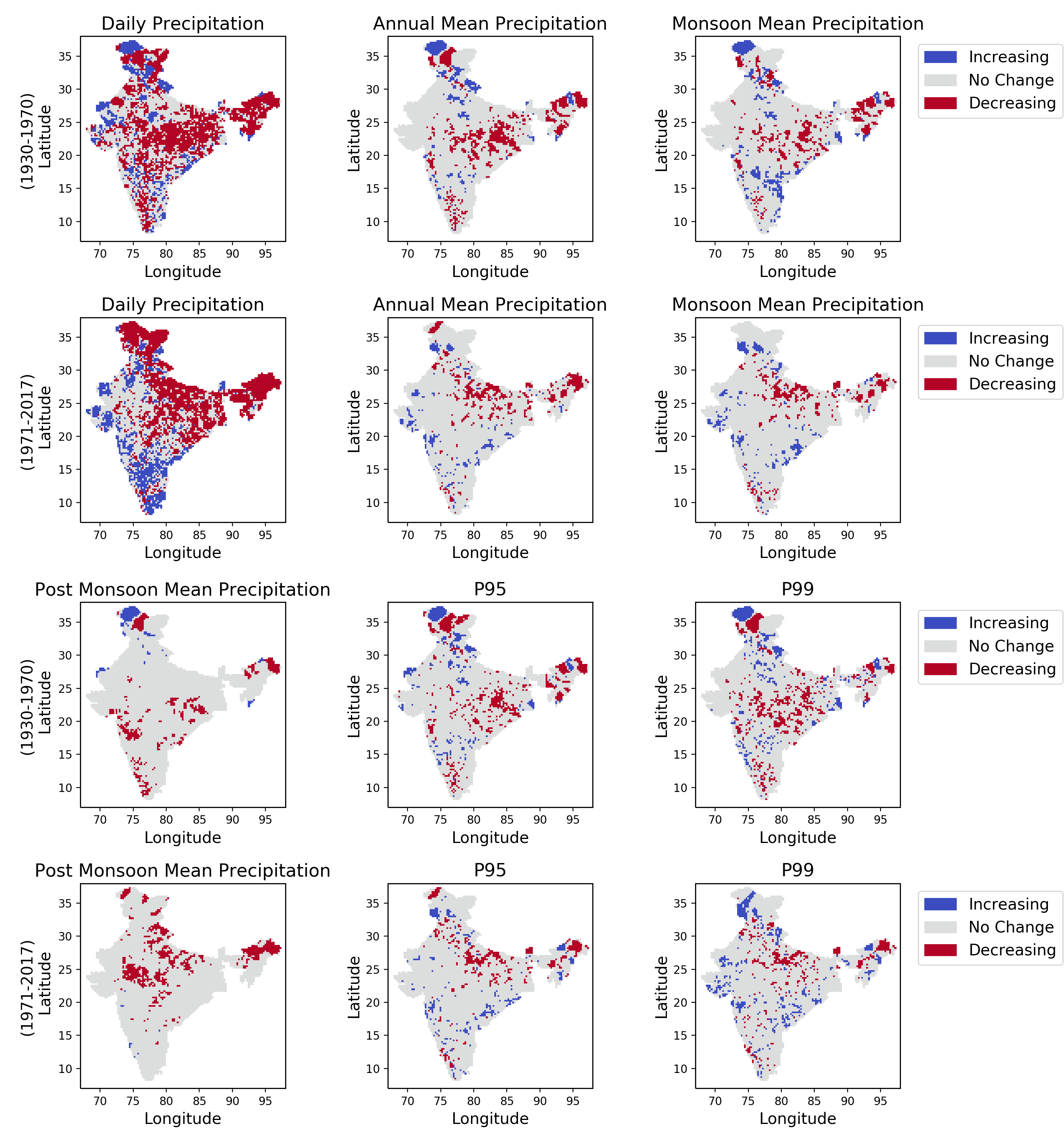


Figure S2: Spatial distribution of significant trends for different statistics related to observed precipitation for 1930-1970 and 1971-2017. Post Monsoon Mean Precipitation refers to the mean daily precipitation during the months after monsoon (October-November). P95 and P99 show the series of 95^th^ and 99^th^ percentile of daily precipitation annually, respectively.


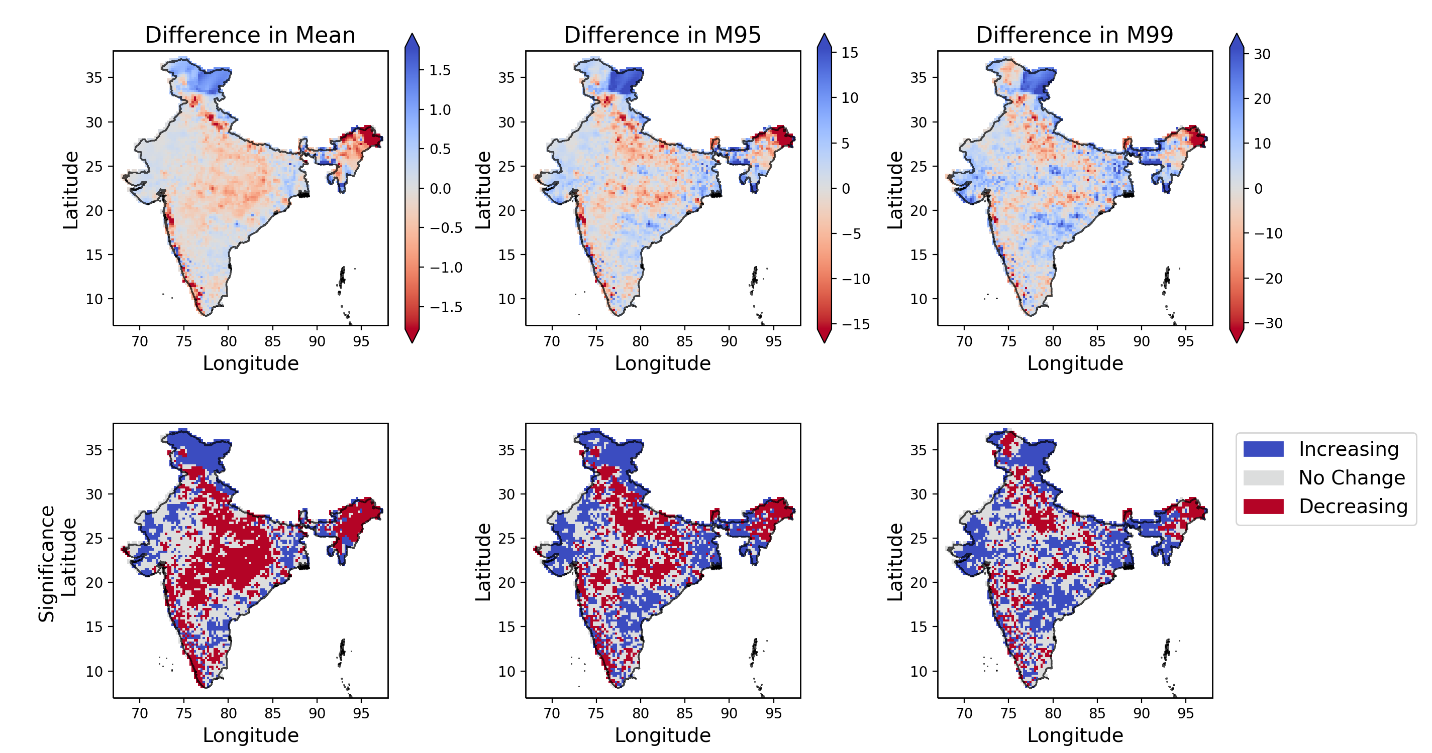


Figure S3: Difference in mean daily precipitation, M95 and M99 (mm/day) for 1971-2017 vs. 1930-1970 and areas marked with nature of significance. M95 is the annual mean precipitation during the days which received more than 95^th^ percentile daily precipitation. Similarly, M99 is the annual mean precipitation during the days which received more than 99^th^ percentile daily precipitation.

Table S1: Details of different CORDEX simulation outputs used in this study

| S.No. | RCM Used | GCM Forcing | Spatial Resolution | Institute |
| --- | --- | --- | --- | --- |
| 1 | REMO2009* | MPI-ESM-LR | 0.5^o^ lat × 0.5^o^ lon | MPI-CSC^+^ |
| 2 | RegCM4 | CCCma-CanESM2 | 50 km by 50 km | IITM^++^ |
| 3 | RegCM4 | CNRM-CM5 | 50 km by 50 km | IITM |
| 4 | RegCM4 | CSIRO-Mk3.6.0 | 50 km by 50 km | IITM |
| 5 | RegCM4 | IPSL-CM5A-LR | 50 km by 50 km | IITM |
| 6 | RegCM4 | MPI-ESM-MR | 50 km by 50 km | IITM |
| 7 | RegCM4 | GFDL-ESM2M | 50 km by 50 km | IITM |

All seven CORDEX models provide output for RCP4.5 and RCP8.5 scenarios.*Scenario RCP2.6 (additionally) available. ^+^ Climate Service Center, Max Planck Institute for Meteorology. *^++^* Indian Institute of Tropical Meteorology

# A1.2 Further details on the spatial analysis using CORDEX model outputs

For studying the future changes in Indian Summer Monsoon rainfall (ISMR), the ensemble mean of CORDEX model outputs is used. First, the ensemble mean CORDEX data is compared with observed precipitation data over Indian landmass to ascertain that whether the ensemble mean CORDEX data represent Indian climatic condition reasonably well. The correlation between monthly mean precipitation from ensemble mean of CORDEX model outputs and observed data is found to be 0.95 when all the months are considered during 1961-2005. However, when only monsoon months (June-September) are considered, the correlation is found to be 0.68 in the same period. Statically significant and high value of the coefficient of correlation suggest that the seasonality of the pan-India precipitation series is represented by CORDEX reasonably well. However, the Indian mainland is climatologically diverse; hence, a spatial analysis of the CORDEX data is also required to check its efficiency in reproducing climatology across India. The spatial variation of daily mean precipitation for the ensemble mean of CORDEX model outputs and observed data is shown in figure S4a-b. Further, the spatio-temporal variation of bias in the ensemble mean of CORDEX model outputs is shown in figure S5. From figure S4c, the positive bias in mean daily precipitation is observed in Northern Himalayan states (Jammu & Kashmir, Ladakh, Himachal Pradesh, and Uttarakhand), and negative bias is observed in the North-Eastern states. Figure S4d shows the spatial distribution of the coefficient of correlation between monthly observed precipitation and monthly ensemble mean of CORDEX simulated precipitation. Barring a few locations (such as Jammu and Kashmir, Ladakh, and some points in south India), the correlation is found to be significant and high. Given the diverse climatic conditions over Indian landmass, the ensemble mean CORDEX data can be assumed to be representing the local climatology reasonably well. It is evident through similar spatio-temporal pattern of mean precipitation and month-wise significant correlation between observed and CORDEX simulated precipitation in most of locations across India. However, the CORDEX simulated precipitation values still have spatially varying bias. To overcome this problem, all the analyses presented in the study (both in the main document and this document) are carried out on the deviation in the future from historical values.

For ensemble mean CORDEX data, the spatial variation for statistically significant changes in different precipitation characteristics/statistics (mean daily precipitation, M95, M99) for different scenarios with respect to their historical values are shown and discussed in figure 2 in main document. The details of the percentage of land under significant statistical change of these precipitation characteristics are tabulated in table S2. From table S2, the land area under significant increase in precipitation characteristics is found to increase progressively between E1 and E3 for RCP 8.5 and RCP4.5. As stated in the manuscript, this intensification is also in terms of magnitude.

A similar spatial intensification pattern is found in case of *REMO2009* CORDEX model forced by MPI-ESM-LR GCM (Figure S6), *RegCM4* CORDEX model driven by CCCma-CanESM2 GCM (Figure S7) and *RegCM4* CORDEX model forced by CSIRO-Mk3.6.0 GCM (Figure S8). The areas showing a high increase are concentrated in the northern Indian Ocean region, south India and south-east Asian countries, as observed in the case of analysis of ensemble mean CORDEX data. Additionally, these is relatively lesser increase in extreme precipitation over the north India. Further, the comparison between figures S4b and S4d, figures S5b and S5d, and figures S6b and S6d suggest that in the case of RCP8.5 the increase in the magnitude of precipitation parameter will be higher than RCP4.5 scenario, indicating the role of anthropogenic activities in the observed changes.

Some possible causes of these observed changes are analyzed in the main document, like change in temperature and precipitable water throughout the future period (2006-2100). The deviation of temperature with their mean historical (1961-2005) values in different epochs for ensemble mean CORDEX is shown in figure S9. Almost all of the study region is found to have statistically significant increase, which amounts to about 2.6 ^o^C and 5 ^o^C increase in some areas for RCP4.5 and RCP8.5 respectively by the end of this century. Most of the study area showing the high increase are land north to 30^o^N (including Himalayan Ranges, Tibetan Plateau, and Pamir ranges). Being a water tower for the region, the increase in temperature in these areas may result in increase in incidents like glacial lake burst and landslide in the region and flood in the downstream. Furthermore, due to a relatively higher increase in temperature over Western Indian Ocean (Near African coast) compared to Eastern Indian Ocean (near Indonesia), the positive Indian Ocean Dipole (pIOD) events is expected to become more frequent with higher intensity. This intensification of pIOD is studied in the subsequent section.

For the analysis of total precipitable water, the output for REMO2009 RCM forced by MPI-ESM GCM is used as other CORDEX models do not provide simulated total precipitable water data. Epochwise changes in total precipitable water is found to be statistically significant throughout the region (Figure S10). However, the areas showing very high increase in temperature (e.g., the Himalayan ranges and Tibetan Plateau) show relatively less increase in total precipitable water content. On the other hand, the Indian Ocean region between 10^o^S –10^o^N is showing very high increase in precipitable water content, which will be strengthening the ISM winds passing through the region.


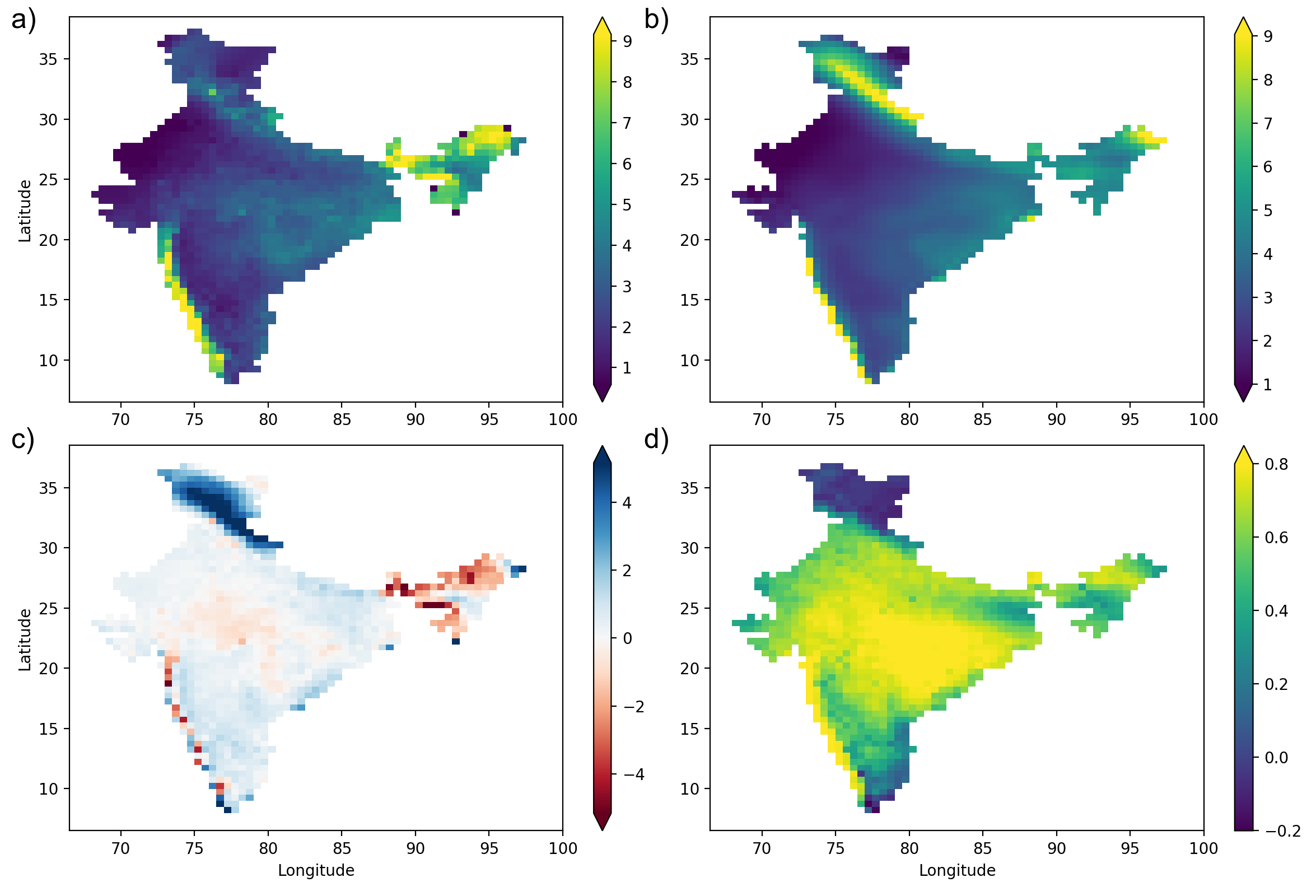


Figure S4: **a)** Mean daily precipitation (mm/day) for observed precipitation during the period 1961-2005, **b)** Mean daily precipitation (mm/day) for mean of CORDEX precipitation ensemble during the period 1961-2005. **c)** The corresponding bias in mean CORDEX precipitation and observed ISMR for daily mean precipitation. **d)** The spatial distribution of the coefficient of correlation calculated between monthly observed precipitation and monthly mean of CORDEX ensemble during 1961-2005.


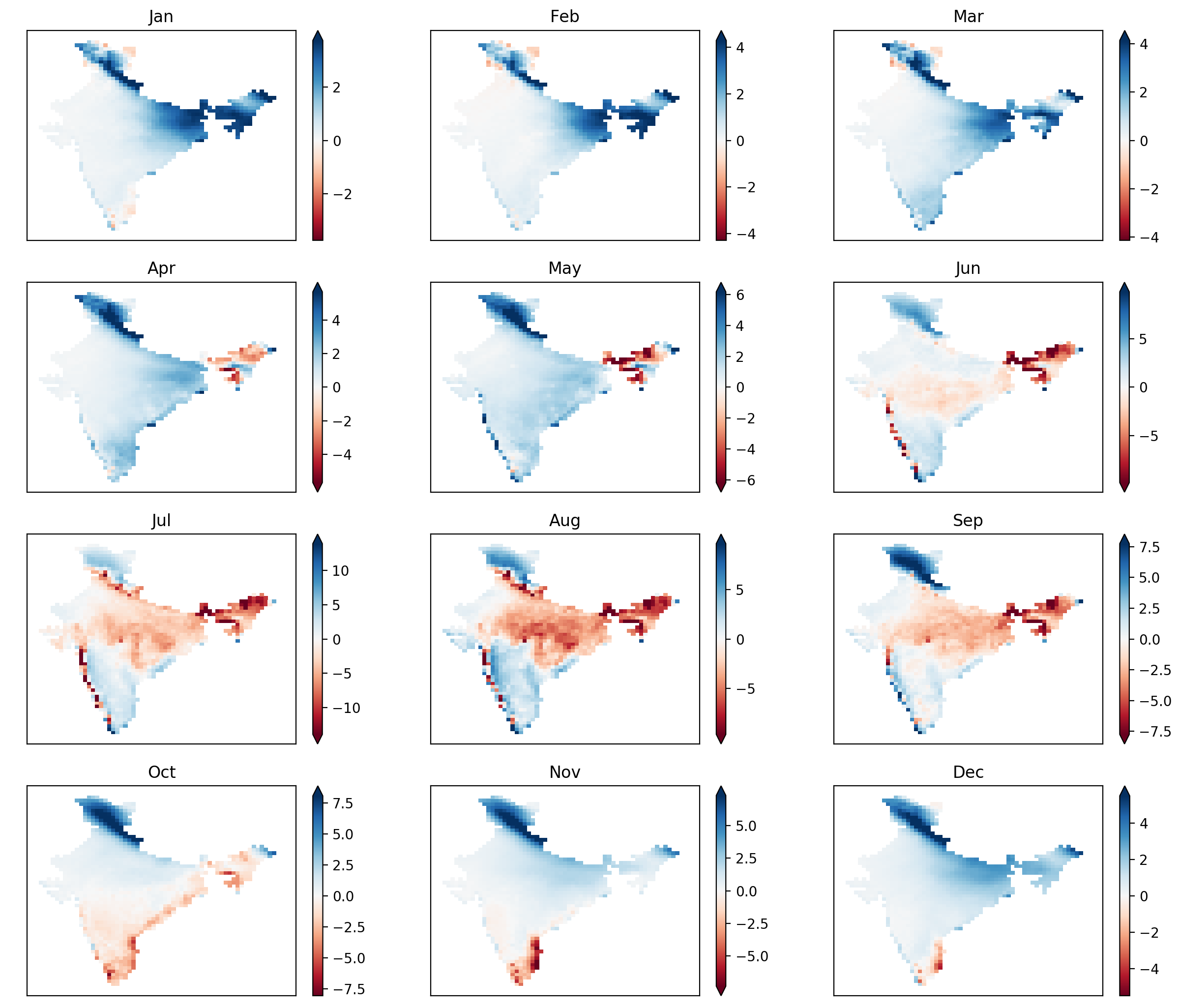


Figure S5: The monthly bias in mean CORDEX precipitation and observed ISMR for daily mean precipitation during 1961-2005.

Table S2: Change in different precipitation statistics (at 5% significance level w.r.t. historical period, 1961-2005) for ensemble mean of CORDEX model outputs.

| **Period** | **Emission Scenario** | **Precipitation Statistics** | **Parameters* of Change in Precipitation Statistics Showing a/an** | | |
| --- | --- | --- | --- | --- | --- |
|  |  |  | **Increase** | **Decrease** | **No change** |
| Entire (2006-2100) | RCP 8.5 | Mean | 84.23  0.6  1.7 | 6.46  0.1  0.2 | 9.31 |
|  |  | M95 | 98.42  3.5  9.9 | 0.15  0.2  0.4 | 1.41 |
|  |  | M99 | 99.23  6.80  18.5 | 0.00  NA  NA | 0.77 |
|  | RCP4.5 | M99 | 95.17  4.20  11.1 | 0.00  NA  NA | 4.83 |
| E1 (2006-2035) | RCP 8.5 | Mean | 66.24  0.2  0.6 | 7.11  0.1  0.3 | 26.65 |
|  |  | M95 | 74.35  1.2  3.2 | 1.05  0.5  1.1 | 24.60 |
|  |  | M99 | 61.93  2.4  5.7 | 1.51  1.5  3.2 | 36.56 |
|  | RCP4.5 | M99 | 54.31  2.3  5.7 | 2.05  1.5  3.6 | 43.64 |
| E2 (2036-2070) | RCP 8.5 | Mean | 82.44  0.5  1.4 | 6.41  0.1  0.3 | 11.15 |
|  |  | M95 | 95.20  2.4  6.7 | 0.43  0.4  1.0 | 4.36 |
|  |  | M99 | 92.96  4.0  10.5 | 0.12  1.8  4.1 | 6.92 |
|  | RCP4.5 | M99 | 82.93  3.1  7.9 | 0.51  1.7  3.3 | 16.55 |
| E3 (2076-2100) | RCP 8.5 | Mean | 83.33  1.1  3.3 | 6.88  0.2  0.4 | 9.79 |
|  |  | M95 | 98.92  6.6  18.9 | 0.09  0.4  0.7 | 0.99 |
|  |  | M99 | 99.69  13.3  36.2 | 0.00  NA  NA | 0.31 |
|  | RCP4.5 | M99 | 96.79  8.0  22.2 | 0.00  1.6  1.6 | 3.21 |

*Three parameters of change (top to bottom in each cell) indicate (i) %age of the study area, (ii) spatially averaged magnitude (in mm/day) of the change and (iii) spatially averaged magnitude (in mm/day) of extreme change (above 95^th^ percentile). NA: Not Applicable.


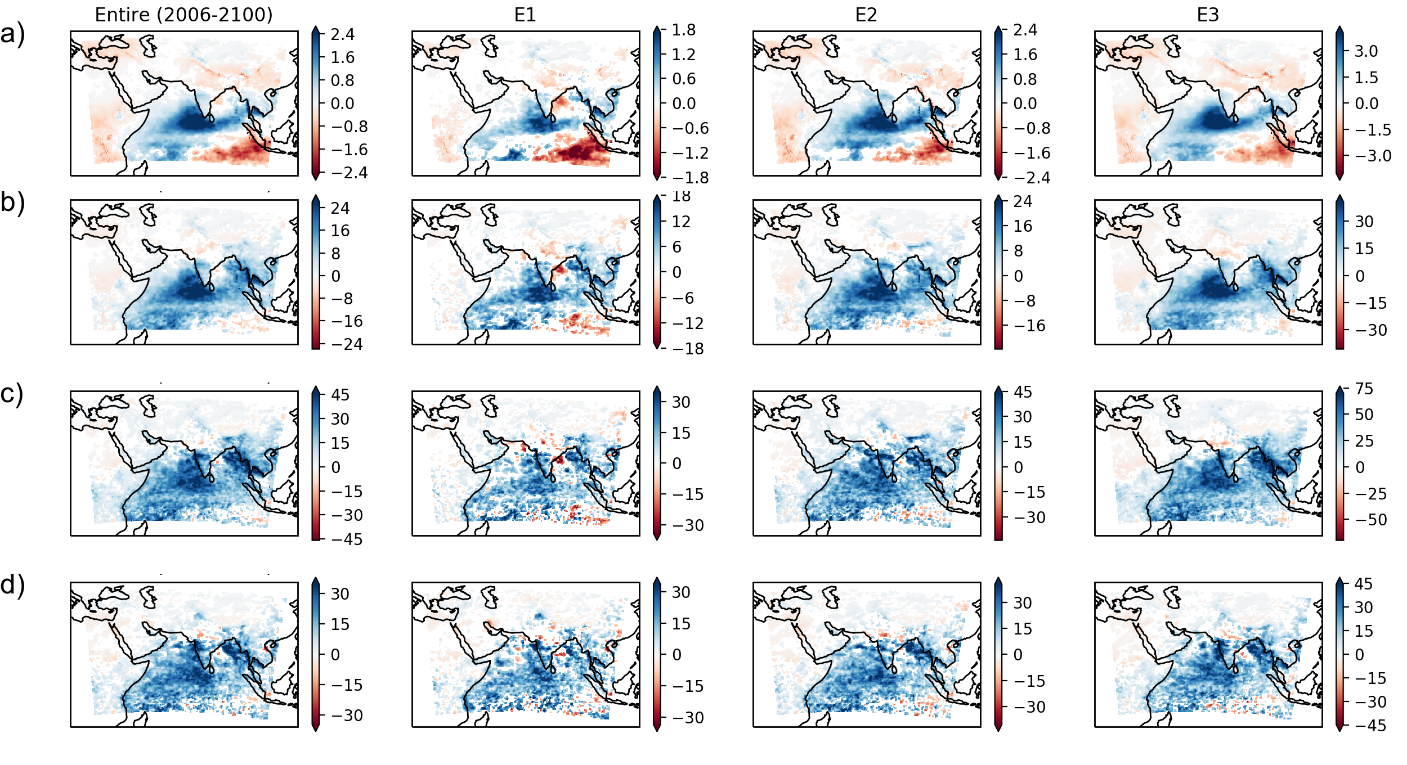


Figure S6: Characteristics of ensemble mean precipitation in future under RCP8.5 and RCP2.6 using REMO2009 simulation driven by MPI-ESM-LR. a-c) Difference in mean, M95 and M99 precipitation (in mm/day) respectively for different periods in future vs. HIST (1961-2005) for RCP8.5 d) Similar to (c) but for RCP2.6. White patches inside the study region show areas with insignificant change. For the definition of the periods (Entire, E1-E3), please refer to figure 2 of the main document.


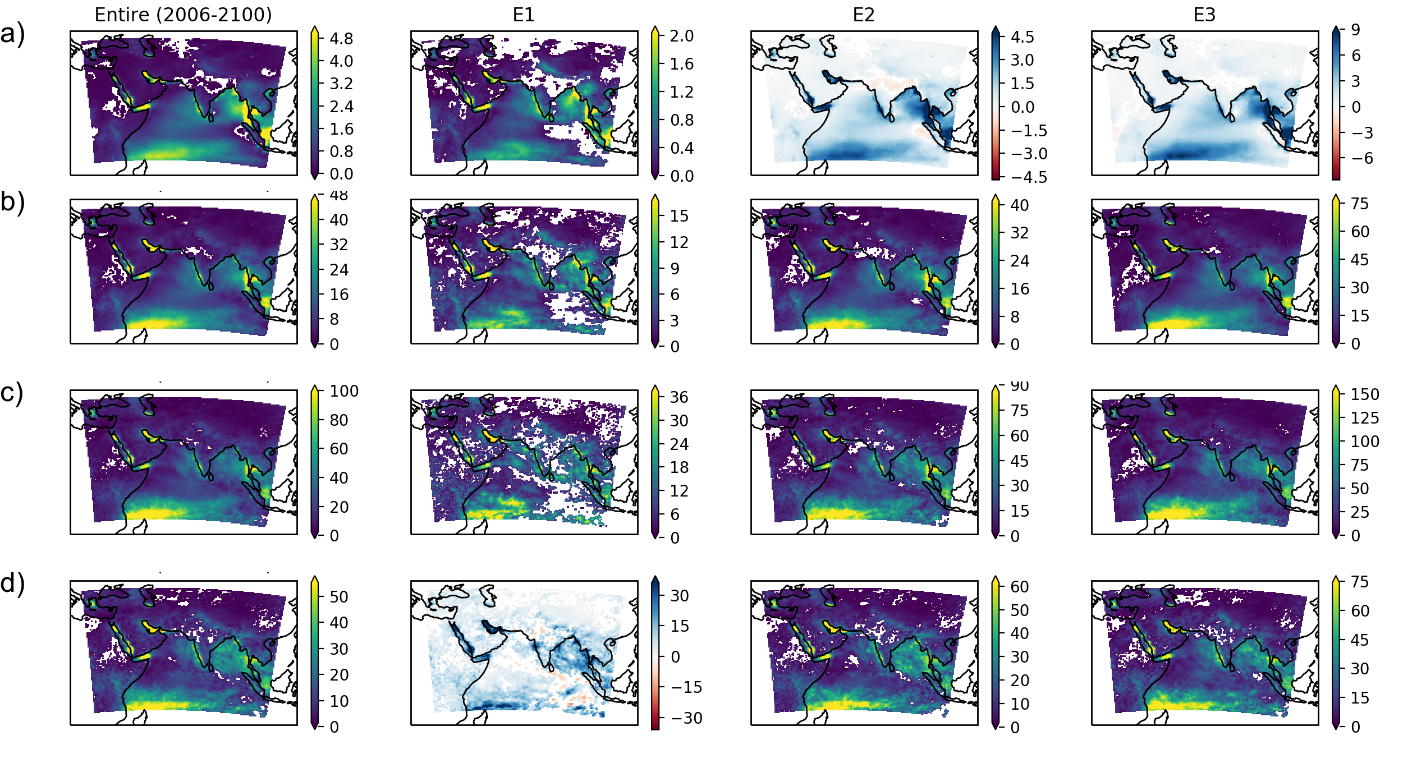


Figure S7: Characteristics of ensemble mean precipitation in future under RCP8.5 and RCP4.5 using RegCM4 simulation driven by CCCma-CanESM2. a-c) Difference in mean, M95 and M99 precipitation (in mm/day) respectively for different periods in future vs. HIST (1961-2005) for RCP8.5 d) Similar to (c) but for RCP4.5. White patches inside the study region show areas with insignificant change.


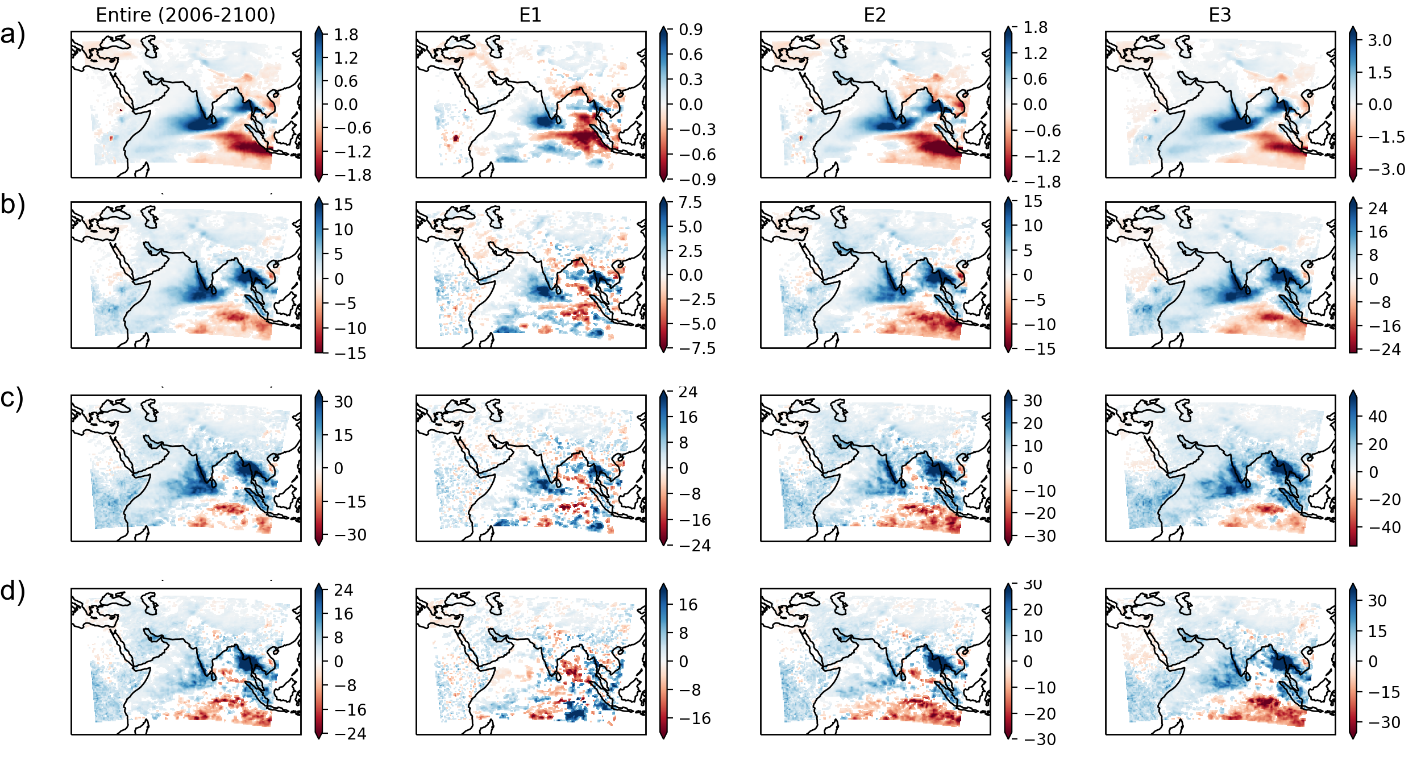


Figure S8: Characteristics of ensemble mean precipitation in future under RCP8.5 and RCP4.5 using RegCM4 simulation driven by CSIRO-Mk3.6.0. a-c) Difference in mean, M95 and M99 precipitation (in mm/day) respectively for different periods in future vs. HIST (1961-2005) for RCP8.5 d) Similar to (c) but for RCP4.5. White patches inside the study region show areas with insignificant change. For the definition of the periods (Entire, E1-E3), please refer to figure 2 of the main document.


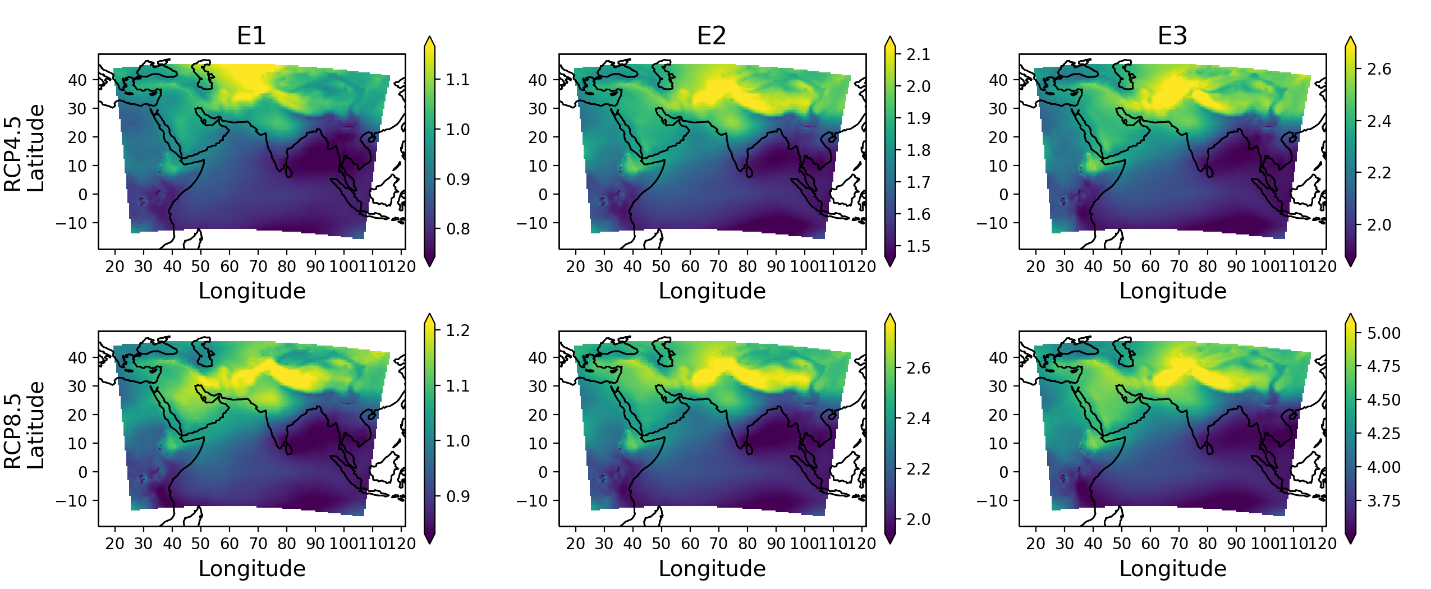


Figure S9: Epochwise change in air temperature (in ^o^C) at 850mb for ensemble mean CORDEX model outputs when compared to historical (1961-2005) mean. There is no region with insignificant change (white patches).


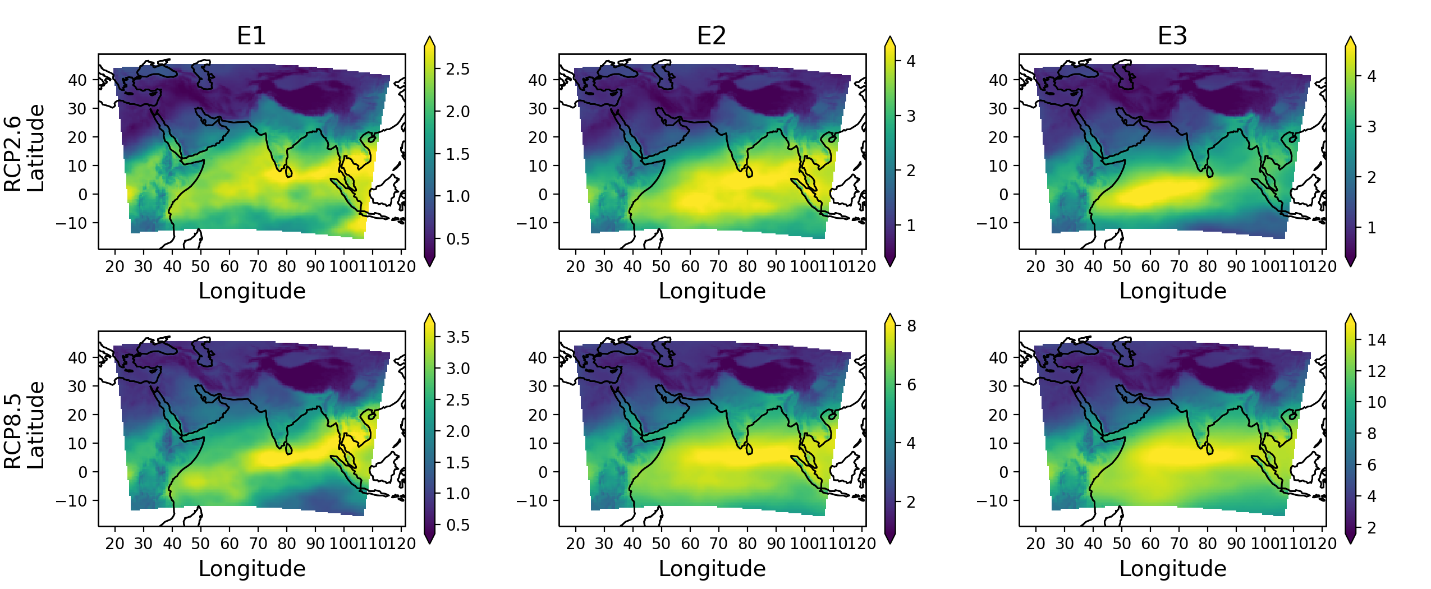


Figure S10: Epochwise change in precipitable water (in kg/m${}^{2}$) for REMO2009 RCM model when compared to historical mean. There is no region with insignificant change (white patches).

# A 1.3 Analysis of observed IOD

As the first epoch period overlaps with observed data available, the result about the expected increase in the number of pIOD can be validated. Dipole Mode Index (DMI) index for IOD (1930-2017) is calculated as difference of standardized sea surface temperature anomaly (base period 1971-2000) over Western Tropical Indian Ocean (WTIO; 10^o^S-10^o^N, 50-70^o^E) and South-Eastern Tropical Indian Ocean (SETIO; 10^o^S-0^o^N, 90-110^o^E) using SST provided by NOAA Extended Reconstructed Sea Surface Temperatures version 5 (ERSSTv5) dataset^4^ with the moving averaging period of 3 months. Weekly DMI index (1982-2017) is also obtained from the State of Ocean Climate Site from NOAA (https://stateoftheocean.osmc.noaa.gov/sur/ind/), which uses NOAA OI.v2 SST (Optimum Interpolation Sea Surface Temperature) dataset^5^. The analysis of the trends in these time series reveals that both WTIO and SETIO anomaly index are having a significant positive trend (tested at 5% level of significance in Mann-Kendall Test) post-1970. The result for NOAA ERSSTv5 data is shown in figure S11. DMI does not show any significant trend for pre and post-1970 periods (tested at 5% level of significance in Mann-Kendall Test). However, despite showing no trend, the difference in mean DMI is found to be statistically significant using Student’s t-test at 5% level of significance during 1971-2017 when compared to 1930-1970. The inter-annual variation for DMI has also increased during post-1970. The variance of DMI during pre- and post-1970 has significantly changed (F test at 5% level of significance). For NOAA ERSSTv5 data, the phase difference between WTIO and SETIO is found to be 3 months. Additionally, peaks (both positive and negative) in DMI are analyzed using the smoothened Z algorithm. In this algorithm, a data point is marked peak when its standardized anomaly calculated using moving mean and moving standard deviation is higher than the selected threshold value. With a moving average window of 2 years and the threshold of 3, the positive IOD events are found to increase more than two times in post-1970 when compared to pre-1970, despite having no significant change in the number of negative IOD events. This intensification of IOD is also suggested by literature^6^.


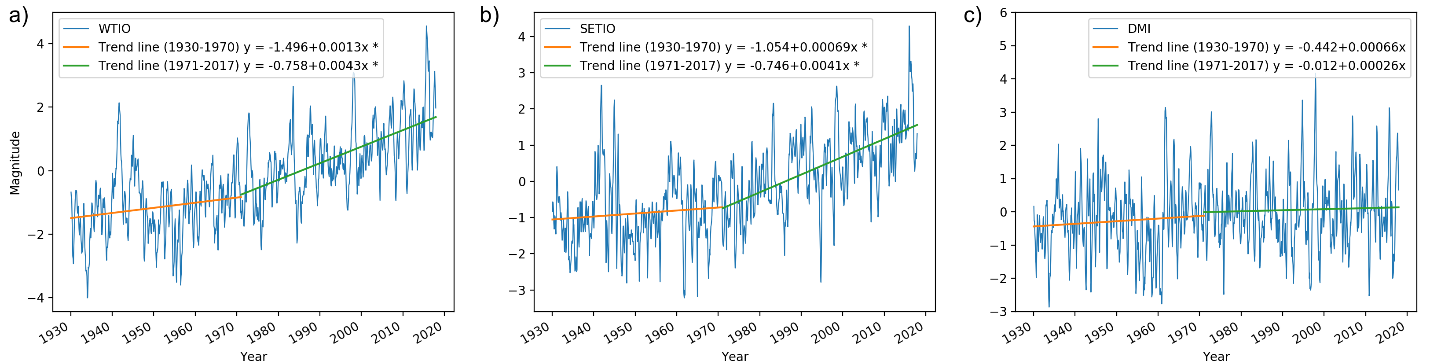


Figure S11: Trend in a) WTIO, b) SETIO, and c) DMI for Indian Ocean Dipole pre and post 1970. The trend line equations marked with * are found to be statistically significant at 5% level of significance using Mann-Kendall’s Test.

# References

1. Suga, T., Kato, A. & Hanawa, K. North Pacific Tropical Water: its climatology and temporal changes associated with the climate regime shift in the 1970s. Progress in Oceanography 47, 223–256 (2000).
2. Savelieva, N., Semiletov, I., Vasilevskaya, L. & Pugach, S. A climate shift in seasonal values of meteorological and hydrological parameters for Northeastern Asia. Progress in Oceanography 47, 279–297 (2000).
3. Gong, D.-Y. & Ho, C.-H. Shift in the summer rainfall over the Yangtze River valley in the late 1970s. Geophysical Research Letters 29, (2002).
4. Huang *et al.* Extended Reconstructed Sea Surface Temperatures Version 5 (ERSSTv5): Upgrades, Validations, and Intercomparisons. *Journal of Climate* (2017)
5. Reynolds *et al.* An improved in situ and satellite SST analysis for climate. *Journal of climate*, *15*(13), pp.1609-1625 (2002).
6. Cai, W. *et al*. Projected response of the Indian Ocean Dipole to greenhouse warming. Nature geoscience **6,** 999 (2013).
